# Supplementary material for: Amidst multiple binding orientations on fork DNA, Saccharolobus MCM helicase proceeds N-first for unwinding
Source: eLife. 2019 Oct 29;8:e46096. doi: 10.7554/eLife.46096 (PMC6831031; doi:10.7554/eLife.46096)
Supplement: Supplementary file 1. [file elife-46096-supp1.docx]

| **Supplementary File 1: DNA Sequences** | |
| --- | --- |
| **DNA** | **Sequence (5’-3’)** |
| DNA129-5 | 5’5GCCTCGCTGCCGTCGCCA |
| DNA164 | 5’TAGGGTCAATAGGTGTAACGAGCTAACTGCGCCGGTCGGGTTGGGTGGGA |
| DNA164-5 | 5’TAGGGTCAATAGGTGTAACGAGCTAACTGCGCCGGTCGGGTTGGGTGGGA5 |
| DNA165 | 5’TCCCACCCAACCCGACCGGCATCTAGTCTGGTAGCGTGAGCGAACGGACC |
| DNA165-3 | 5’3TCCCACCCAACCCGACCGGCATCTAGTCTGGTAGCGTGAGCGAACGGACC |
| DNA171 | 5’CTAACTGCGCCGGTCGGGTTGGGTGGGA |
| DNA172 | 5’TCCCACCCAACCCGACCGGCATCTAGTC |
| DNA180-5 | 5’TGACGTCGCACACCGTGCTC5 |
| DNA188 | 5’GAGCACGGTGTGCGACGTCA |
| DNA189 | 5’GCCGGTCGGGTTGGGTGGGA |
| DNA190 | 5’TCCCACCCAACCCGACCGGC |
| DNA14-B | 5’CGATGAGAGCGAGTCGCATGGTATCGTCBAGCCGGTCGGGGTGGGTGGGAAGCGTAGGGAGAGGTG |
| DNA179-F | 5’GGATGCGAAGGGTGGGTGGGCGACCGGCTTGACGAFACCATGCGACTCGCTCTCATCG |
| DNA182-F | 5’GGGTGGGCGACCGGCTTGACGAFACCATGCGACTCGCTCTCATCG |
| DNA60-F | 5’FGCGAGGCGAGCGCGAGCG |
| DNA202-B | 5’CGCTCGCGCTCGCCTCGCATCTGBCATGGTATTCTGATTGATAGTAGCCGAGAGTAGTGAGAGTAGCTAGAGAGTATTCACAGCGGTCAGCTCGTGCAGC |
| DNA203-B | 5’CGCTCGCGCTCGCCTCGCATCTGTCATGGTATTCTGATTGATAGTAGCCGAGAGTAGTGAGAGTAGCTAGAGAGTABTCACAGCGGTCAGCTCGTGCAGC |
| DNA204-F | 5’GCTGCACGAGCTGACCGCT­F |
| G452C For | 5'GCAGGGAATCCGAAATTCTGTAGATACATAAGTGAAAGGCCTGTGTCTGATAATATCAAC |
| G452C Rev | 5'GTTGATATTATCAGACACAGGCCTTTCACTTATGTATCTACAGAATTTCGGATTCCCTGC |
| G456C For | 5'GGAATCCGAAATTCGGGAGATACATATGTGAAAGGCCTGTGTCTGATAATATCAACCTAC |
| G456C Rev | 5'GTAGGTTGATATTATCAGACACAGGCCTTTCACATATGTATCTCCCGAATTTCGGATTCC |
| 5 – Cy5, 3 – Cy3, F – fluorescein (FAM), B - Biotin | |
